# Efficacy of *Acacia nilotica* aqueous extract in treating biofilm-forming and multidrug resistant uropathogens isolated from patients with UTI syndrome

**DOI:** 10.1038/s41598-020-67732-w

**Published:** 2020-07-07

**Authors:** Rokaia B. Elamary, Fawziah M. Albarakaty, Wesam M. Salem

**Affiliations:** 1 Department of Botany and Microbiology, Faculty of Science, South Valley University, Qena, 83523 Egypt; 2 Department of Biology, College of Applied Sciences, Umm Al Qura University, Makkah Al Moukarramh, Saudi Arabia; 3 Department of Biology, College of Science, Princess Nourah Bint Abdul Rahman University, Riyadh, Saudi Arabia

**Keywords:** Microbiology, Molecular biology

## Abstract

*Escherichia coli* is the dominant bacterial cause of UTI among the uropathogens in both developed and developing countries. This study is to investigate the effect of *Acacia nilotica* aqueous extract on the survival and biofilm of isolated pathogens to reduce UTIs diseases. A total of 170 urine samples were collected from Luxor general hospital and private medical analysis laboratories in Luxor providence, Egypt. Samples were screened for the incidence of uropathogens by biochemical tests, antibiotics susceptibility, detection of virulence, and antibiotic-resistant genes by multiplex PCR, biofilm formation, and time-killing assay. *Escherichia coli* is by far the most prevalent causative agent with the percentage of 73.7% followed by *Klebsiella pneumoniae*, *Proteus mirabilis*, *Pseudomonas aeuroginosa,* and *Acinetobacter baumanii*. Isolates were multidrug-resistant containing *bla*_TEM_, *bla*_SHV_, *bla*_CTX_, *qnrs,* and *aac*(3)-Ia resistant genes. All isolates were sensitive to 15–16.7 mg ml^−1^ of *Acacia nilotica* aqueous extract. Time killing assay confirmed the bactericidal effect of the extract over time (20–24 h). A high percentage of 3-Cyclohexane-1-Carboxaldehyde, 2,6,6-trimethyl (23.5%); á-Selinene (15.12%); Oleic Acid (14.52%); Globulol (11.35%) were detected among 19 bioactive phytochemical compounds in the aqueous extract of *A. nilotica* over the GC-mass spectra analysis. The plant extract reduced significantly the biofilm activity of *E. coli*, *K. pneumoniae*, *P. mirabilis,* and *P. aeuroginosa* by 62.6, 59. 03, 48.9 and 39.2%, respectively. The challenge to improve the production of *A. nilotica* phytochemicals is considered a very low price for the return.

## Introduction

Urinary tract infections (UTI_S_) are one of the most prevalent and predominant nosocomial human infections. It infects patients of all ages and both gender with the greatest occurrence in females^1^. Signs and symptoms may include fever, chills, dysuria, urinary urgency, frequency, and cloudy or malodorous urine^2^. UTIs are caused by a variety of bacteria such as *E. coli*, *K. pneumoniae*, *P. mirabilis*, *Pseudomonas* sp., *S. aureus*, *Enterococcus faecalis*, *Streptococcus* sp., and *Citrobacter* sp.^3^. Each organism has its virulence genes that contribute to its invasion and toxicity. The increasing prevalence of UTI and antibiotic-resistant bacteria have made empirical antibiotic treatment more and more difficult^4^. A urine culture and antibiotic susceptibility tests are important for diagnosing the disease, recommending suitable antibiotics, and reducing the number of antibiotic-resistant uropathogens^2^. On the other hand, the genus *Acacia* belongs to the Leguminosae family. It contains more than 1,350 species, distributed throughout tropical and warm areas^5^. Several species of *Acacia* have been proven as significant antibacterial and antifungal agents^6,7^. It also has a great effect against multidrug-resistant strains of bacteria initiating nosocomial and community-acquired infections^8^. The plant is a tree with yellow mimosa-like flowers and long grey pods^9^. The use of plants and herbs extract in the therapy of human disease is very ancient traditions and scientists in Africa and other developing countries are carrying research on local plants numerous in the continent for use in conventional medicine^10^. The current study was performed to determine the resistant patterns of uropathogens and to highlight the efficacy of phytochemical compounds in *Acacia nilotica* aqueous extract against the survival and biofilm of these pathogens to reduce urinary tract infection diseases.

## Results

### The incidence of uropathogenic bacteria among examined urine samples

The prevalence of the isolated uropathogens in urine samples were illustrated in Table 1. Among the 170 urine samples, only 133 (78.2%) sample was positive for urine culture. Positive samples comprise 32 (53.3%) samples from males and 101 (91.8%) from females. The most common prevalent organism (from each corresponding positive samples) was *E. coli* which isolated from 98 patient with a percentage of 73.7%, followed by *K. pneumoniae* 13.5% (18), *P. mirabilis* 6.7% (9), *P. aeruginosa* 4.5% (6) and *A. baumannii* 1.5% (2). The total bacterial count of all samples was ranged from 1.88 to 215 × 10^7^ CFU/ml.

**Table 1:** Incidence of isolated uropathogenic bacteria in urine samples.

Parameters	No. of positive isolates^b^	(%)^c^	CFU/ml^d^
Isolates^a^			
*Escherichia coli*	98	73.7	215 × 10^7^
*Klebsiella pneumoniae*	18	13.5	16.8 × 10^7^
*Proteus mirabilis*	9	6.7	111 × 10^7^
*Pseudomonas aeuroginosa*	6	4.5	1.88 × 10^7^
*Acinetobacter baumanii*	2	1.5	202 × 10^7^
Total	133		

Total number of examined urine samples was 170 sample, they were 110 sample from females and 60 samples from male. Number of positive samples was 133 samples comprised from 101 sample from females and 32 sample from males.

^a^Different isolated uropathogens from urine samples.

^b^Number of positive isolates from total number of positive samples.

^c^Percentage of positive isolates from total number of positive samples.

^d^Colony forming unit per ml for each isolated bacterium.

### Antimicrobial susceptibility testing

The rate of resistance to all isolated uropathogenic bacteria to a panel of antibiotics with different potency was illustrated in Table 2, the resistance rate of ampicillin-sulbactam, ampicillin, gentamicin, nalidixic acid, amikacin, ceftazidime, ciprofloxacin, piperacillin, and cefepime was observed in all isolated uropathogens. While resistance level of piperacillin-tazobactam was noticed in all isolated pathogens except *P. aeruginosa* that was sensitive for this antibiotic. Interestingly, sensitivity level imipenem and meropenem were observed only against *E. coli* and *P. mirabilis*. So, antimicrobial susceptibility demonstrated that *K. pneumoniae* and *A. baumannii* were resistant to all tested antibiotics (100%). Followed by *P. aeruginosa* that was resistant to 91.6% of all tested antibiotics. Finally, *E. coli* and *P. mirabilis* were resistant to 83.3% of antibiotics**.**

**Table 2:** Antimicrobial susceptibility and antibiotic resistant genes of multidrug resistant uropathogens isolated from urine.

Isolates^a^	*E. coli*	*K. pneumoniae*	*P. mirabilis*	*P. aeruginosa*	*A. baumannii*
Antibiotics^b^					
SAM	R	R	R	R	R
AM	R	R	R	R	R
GM	R	R	R	R	R
NA	R	R	R	R	R
AMK	R	R	R	R	R
CAZ	R	R	R	R	R
CIP	R	R	R	R	R
PIP	R	R	R	R	R
PTZ	R	R	R	**S**	R
CPE	R	R	R	R	R
MEM	**S**	R	**S**	R	R
IPM	**S**	R	**S**	R	R
Antibiotic resistant genes^c^					
*bla*_TEM_	+	–	+	–	+
*bla*_*SHV*_	+	+	+	+	+
*bla*_*CTX*_	+	–	–	+	+
*qnrS*	+	+	+	+	+
*aac(3)-Ia*	+	+	+	+	+
*mex*R^**d**^	—	—	—	+	—

^a^*Escherichia coli, Klebsiella pneumoniae, Proteus mirabilis, Pseudomonas aeuroginosa, Acinetobacter baumanii.*

^b^SAM (Ampicillin-sulbactam), AM (Ampicillin), GM (Gentamicin), NA (Nalidixic acid), AMK (Amikacin), CAZ (Ceftazidime), CIP (Ciprofloxacin), PIP (Piperacillin), PTZ (Piperacillin-tazobactam), CPE (Cefepime), MEM (Meropenem), IPM (Imipenem). R = Resistant; S = Sensitive.

^c^Antibiotic resistant genes,+: present; –: absent.

^d^*mex*R gene: it is specific gene for *Pseudomonas aeuroginosa* only.

### Detection of virulence and antibiotic-resistant genes

**Figure 1:**
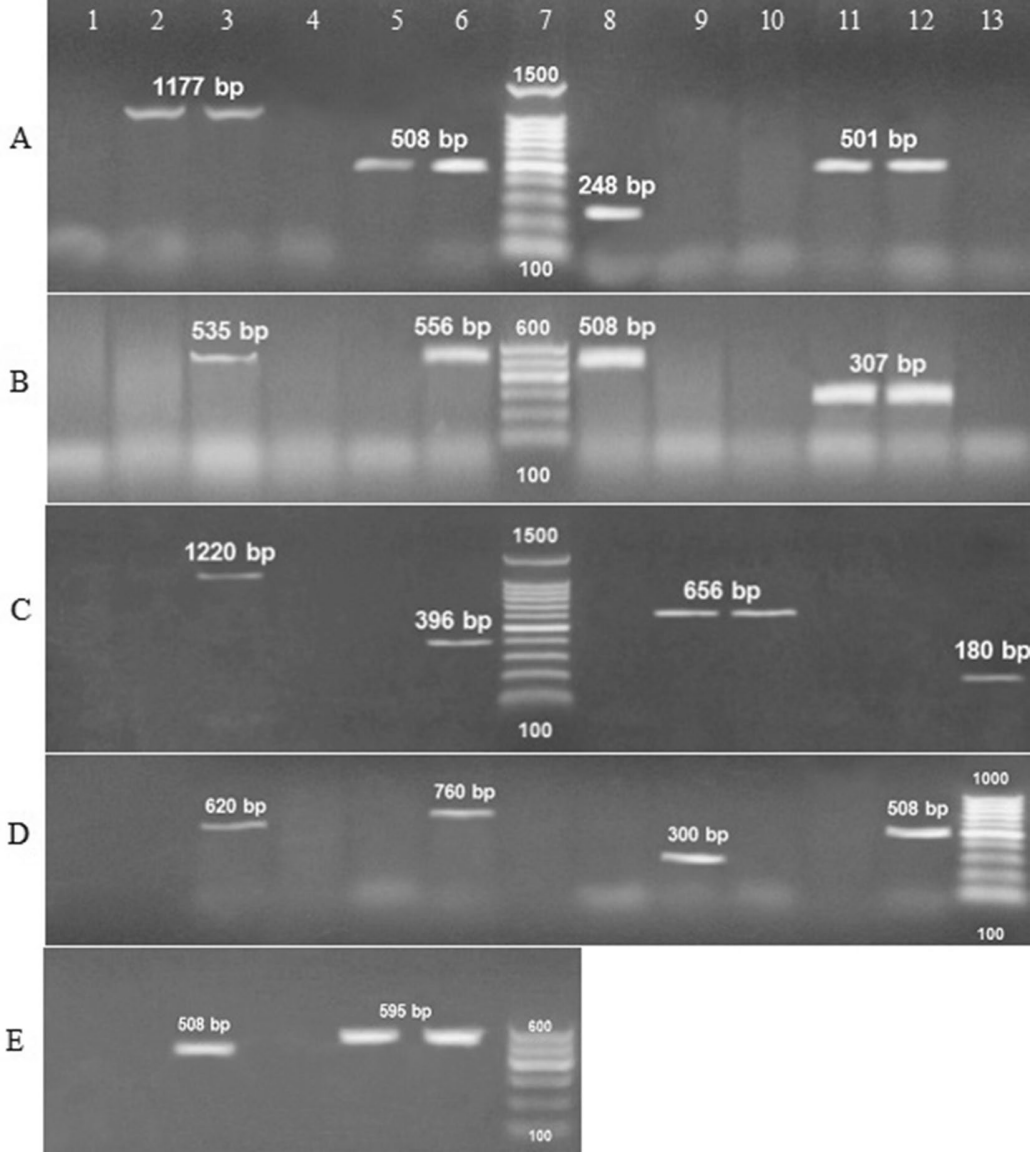
1.5% agarose gel electrophoresis of multiplex PCR of virulence genes characterized for the isolated uropathogens. (**A**) *E. coli*; Lane 1, 4, 10, 13: negative control for detected genes; Lane 3, 6, 8, 11 positive control of DNA confirmed by reference laboratory for quality control; Lane 2 *hly* (1,177 bp), Lane 5 *fim*H (508 bp), Lane 9 negative *eae*A (248), Lane 12 *pap*C (501 bp); Lane 7 Gel Pilot 100 bp plus ladder (cat. no. 239045) supplied from QIAGEN (USA). (**B**) *Klebsiella pneumoniae*; Lane 1, 4, 10, 13: negative control for detected genes; Lane 3, 6, 8, 11 positive control of DNA; Lane 2 negative *rmp*A (535 bp), Lane 5 negative *Tra*T (556 bp), Lane 9 negative *fim*H (508), Lane 12 aerobactin (307 bp); Lane 7, 100 bp ladder as molecular size DNA marker (cat. no. 239035) supplied from QIAGEN (USA). (**C**) *Pseudomonas aeruginosa*; Lane 1, 4, 8, 11: negative control for detected genes; Lane 3, 6, 10 and 13: positive control of DNA; Lane 2 negative *las*B (1,220 bp); Lane 5 negative *tox*A (396); Lane 9 *psl*A (656 bp); Lane 12 negative *fli*C (180 bp); Lane 7 Gel Pilot 100 bp plus ladder (cat. no. 239045) supplied from QIAGEN (USA). (**D**) *Acinetobacter baumanii;* Lane 1, 4, 7, 10: negative control for detected genes; Lane 3, 6, 9 and 12: positive control of DNA; Lane 2 negative *cnf*1 (620 bp); Lane 5 negative *cva*C (760); Lane 8 negative *iut*A (300 bp); Lane 11 negative *fim*H (508 bp); Lane 13 Gene ruler 100 bp DNA ladder (cat. no. SM0243) supplied from Fermentas. (**E**) *Proteus mirabilis;* Lane 1 and 4: negative control for detected genes; Lane 3 and 6: positive control of DNA; Lane 2 negative *fim*H (508 bp); Lane 5 *atp*D (595 bp); Lane 7, 100 bp ladder as molecular size DNA marker (cat. no. 239035) supplied from QIAGEN (USA).

**Figure 2:**
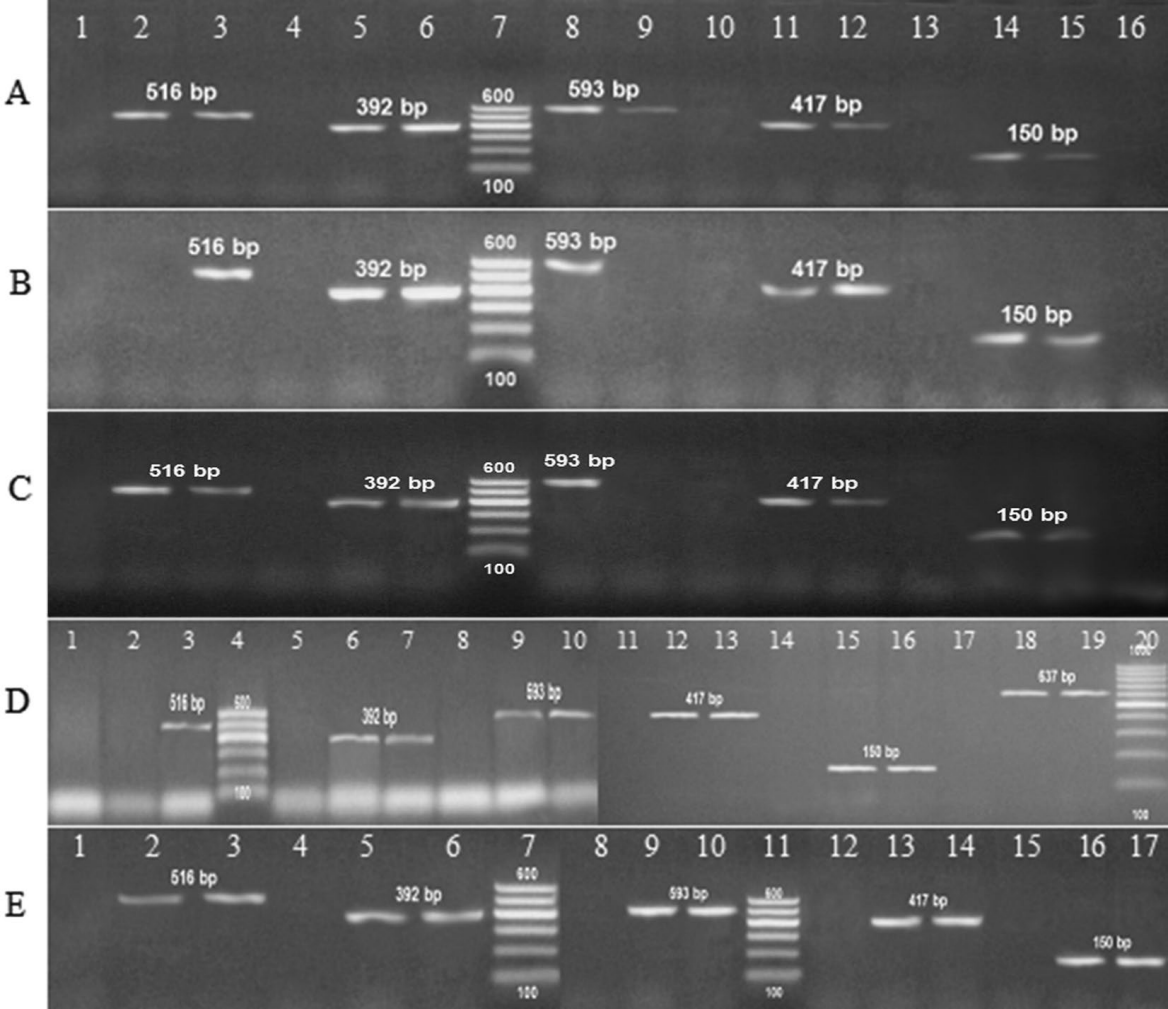
1.5% agarose gel electrophoresis of multiplex PCR of antibiotic resistant genes for the isolated uropathogens. (**A**) *E. coli*; (**B**) *Klebsiella pneumoniae*; (**C**) *Proteus mirabilis*; Lane 1, 4, 10, 13 and 16: Negative control for detected genes; Lane 3, 6, 8, 11 and 14: Positive control of DNA confirmed by reference laboratory for quality control; Lane 7: 100 bp ladder as molecular size DNA marker (cat. no. 239035) supplied from QIAGEN (USA). Lane 2 *bla*_TEM_ (516 bp); Lane 5 *bla*_SHV_ (392 bp); Lane 9 *bla*_CTX_ (593 bp); Lane 12 *qnr*S (417); Lane 15 *aac*(3)-Ia (150 bp). (**D**) *Pseudomonas aeuroginosa*; Lane, 1, 5, 8, 11, 14 and 17: Negative control for detected genes; Lane 3, 7, 10, 13, 16 and 19: Positive control of DNA; Lane 2 *bla*_TEM_; Lane 6 *bla*_SHV_; Lane 9 *bla*_CTX_; Lane 12 *qnr*S; Lane 15 *aac*(3)-Ia; Lane 18 *mex*R (637 bp). Lane 4: 100 bp ladder as molecular size DNA marker (cat. no. 239035) supplied from QIAGEN (USA). Lane 20: Gene ruler 100 bp DNA ladder (cat. no. SM0243) supplied from Fermentas. (**E**) *Acinetobacter baumanii;* Lane 1, 4, 8, 12, 15: Negative control for detected genes; Lane 3, 6, 10, 14 and 17: Positive control of DNA; Lane 2 *bla*_TEM_; Lane 5 *bla*_SHV_; Lane 9 *bla*_CTX_; Lane 13 *qnr*S; Lane 16 *aac*(3)-Ia. Lane 7 and 11: 100 bp ladder as molecular size DNA marker (cat. no. 239035) supplied from QIAGEN (USA).

The multiplex PCR screening for virulence and antibiotic-resistant genes showed that *hly*, *pap*C, and *fim*H virulence genes were present *E. coli* while *eae*A was absent. For *K. pneumoniae*, *aerobactin* gene was present while, *rmp*A, *Tra*T, and *fim*H were absent. For the *P. mirabilis atp*D gene was a present while, the *fim*H gene was absent. For *P. aeruginosa*, the *psl*A gene was present while, *las*B, *tox*A, and *fli*C were absent. Finally, *A. baumannii* doesn't contain any of the detected genes (*cnf*1, *cva*C, *iut*A, and *fim*H) (Table 3; Fig. 1). On the other hand, the antibiotic-resistant genes, *bla*_SHV_, *qnr*S, and *aac*(3)-Ia were present in all uropathogenic isolates (100%). *bla*_TEM_ was present among *E. coli*, *P. mirabilis,* and *A. baumannii* with a percentage of 100% and absent in other isolates. *Bla*_CTX_ gene was detected among *E. coli*, *P. aeruginosa* and *A. baumannii* while they were absent in other isolates. Finally, *mex*R was positive among *P. aeruginosa* isolates (Table 2; Fig. 2).

**Table 3:** The relation between virulence genes and antibacterial activity of *Acacia nilotica* extract for isolated uropathogens.

Isolates^a^	Virulence genes^b^				Antibacterial efficacy
					MIC/MBC^c^ (mg ml^−1^)
*E. coli*	*hly*	*fim*H	*eae*A	*pap*C	11.7/13.3
	+	+	–	+	
*K. pneumoniae*	*rmp*A	*aerobactin*	*fim*H	*Tra*T	11.7/13.3
	–	+	–	–	
*P. mirabilis*	*fim*H		*atp*D		15/16.7
	–		+		
*P. aeruginosa*	*las*B	*tox*A	*psl*A	*fli*C	11.7/15
	–	–	+	–	
*A. baumanii*	*cnf*1	*cva*C	*iut*A	*fim*H	11.7/15
	–	–	–	–	

^a^Different isolated bacteria from urine samples; *Escherichia coli*, *Klebsiella pneumoniae*, *Proteus mirabilis*, *Pseudomonas aeruginosa*, *Acinetobacter baumannii.*

^b^Present ( +), absent (–).

^c^Minimal inhibitory concentration/ minimal bactericidal concentration of the extract against isolated uropathogens represented in mg ml^−1^.

### GC–MS analysis

**Figure 3:**
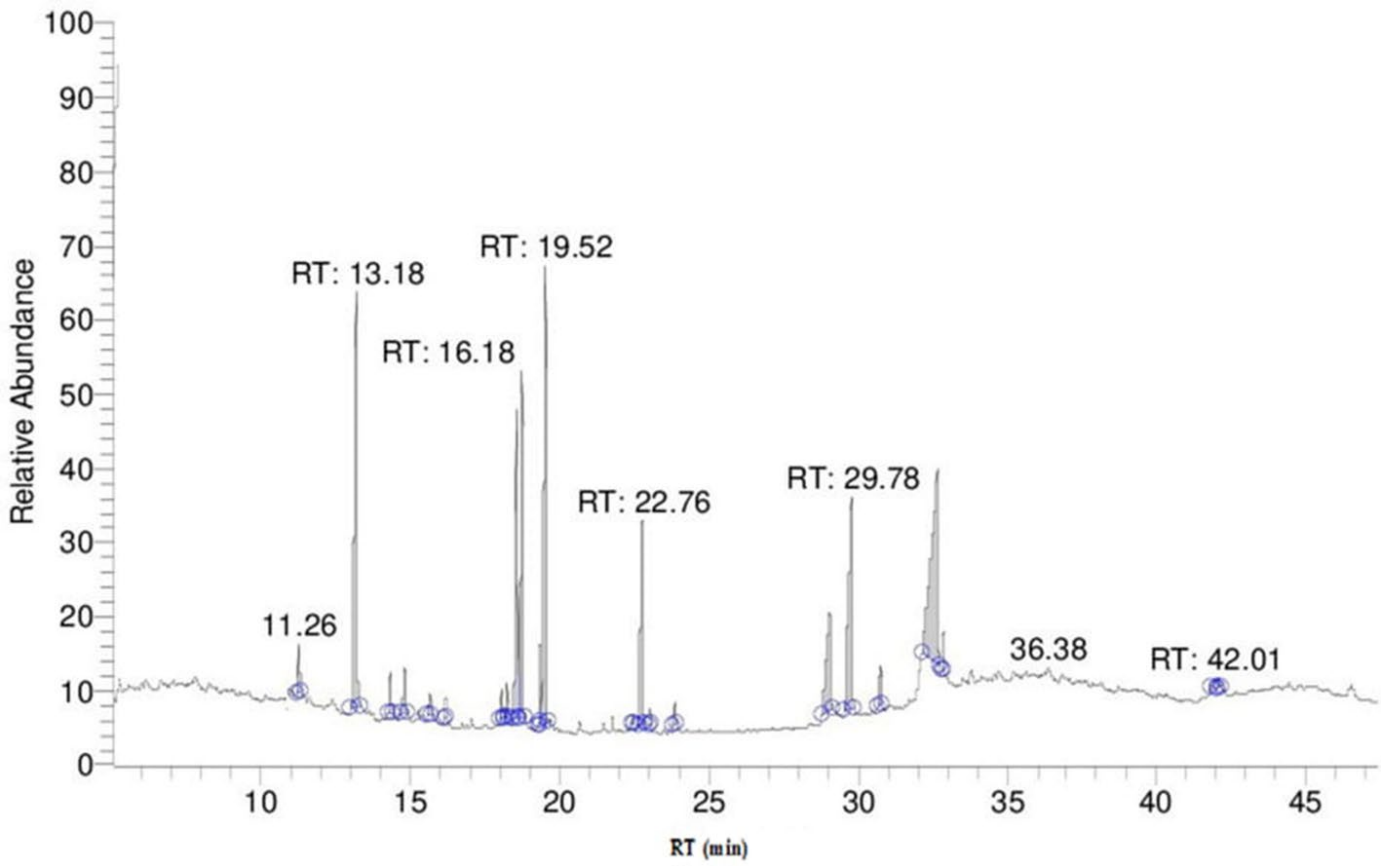
Gas chromatography spectra of biologically active compounds of *Acacia nilotica*.

The analysis and extraction of plant material play a significant role in the progress, reconstruction, and quality control of herbal formulations. Hence one of the important aims in the present study was to find out the bioactive compounds present in the aqueous extract of *A. nilotica* by using Gas chromatography-Mass spectroscopy. This shows the presence of 19 bioactive phytochemical compounds in the aqueous extract of *A. nilotica*. The highest percentage content of the compounds are as follows: 3-Cyclohexane-1-Carboxaldehyde, 2,6,6-trimethyl (23.5%); á-Selinene (CAS) (15.12%); Oleic Acid (14.52%); Globulol (11.35%). Other active compounds with their peak number, concentration (peak area%), and retention time (RT) are presented in (Table 4; Fig. 3).

**Table 4:** GC-mass spectra of *Acacia nilotica* showing different active compounds.

S. no.	RT (min)	Compound	Area (%)	M. formula	M. wt
1	11.26	Carane, 4,5-epoxy-, (E)-	0.86	C_10_H_16_O	152
2	13.18	3-Cyclohexane-1-Carboxaldehyde, 2,6,6-trimethyl-	23.5	C_10_H_16_O	152
3	14.33	Cycloundecene, 1-methyl-	0.76	C_12_H_22_	166
4	14.80	2,4-Decadienal, (E, E)-(CAS)	1.05	C_10_H_16_O	152
5	15.63	1-Decanol, 2-methyl-	0.47	C_11_H_24_O	172
6	16.18	Cyclohexane,1-ethyl-1-methyl-2,4-bis(1-methyl)-(CAS)	0.72	C_15_H_24_	204
7	18.06	á-Chamigrene	0.52	C_15_H_24_	204
8	18.21	á-Guaiene	0.59	C_15_H_24_	204
9	18.57	á-Selinene (CAS)	15.12	C_15_H_24_	204
10	19.32	1,3-Benzodioxole, 4-methoxy-6-(2-propenyl)-	1.05	C_11_H_12_O_3_	192
11	19.52	Globulol	11.35	C_15_H_26_O	222
12	22.76	1,3-Benzodioxole, 4,7-dimethoxy-5-(2-propeny1)-(CAS)	5.54	C_12_H_14_O_4_	222
13	23.02	a-acorenol	0.18	C_15_H_26_O	222
14	23.86	1,2-Epoxycyclooct-3-ene, 5,5-dimethyl-8-methylene-	0.32	C_11_H_16_O	164
15	29.09	Hexadecanoic acid (CAS)	4.59	C_16_H_32_O_2_	256
16	29.78	26,8a-(Dimethoxy)-3,5,7-trion tetracyclo [7.2.1.0(4,11).0(6,10)] dodecane	8.51	C_11_H_16_O_5_	228
17	30.75	Isobergapten	1.09	C_12_H_80_O	216
18	32.66	Oleic acid	14.52	C_18_H_34_O_2_	282
19	32.86	Isochiapin B	1.18	C_19_H_26_O_6_	346

RT: Retention time per minute; active compounds detected by GC mass; area (%): percentage of compound; M. formula: molecular formula; M. wt: molecular weight of the compound.

**Table 5:** Primers sequences, target genes, amplicon sizes and cycling conditions.

Target gene	Sequence		Amplified segment (bp)		Primary denaturation		Amplification (35 cycles)					References
							Secondary denaturation	Annealing	Extension	Final extension		
Virulence genes used for *E. coli* isolates												
*fim*H	TGCAGAACGGATAAGCCGTGG GCAGTCACCTGCCCTCCGGTA		508		94 °C/5 min		94 °C/30 s	50 °C/40 s	72 °C/45 s	72 °C/10 min		^81^
*hly*	AACAAGGATAAGCACTGTTCTGGCT ACCATATAAGCGGTCATTCCCGTCA		1,177		94 °C/5 min		94 °C/30 s	60 °C/40 s	72 °C/1 min	72 °C/12 min		^82^
*pap*C	TGATATCACGCAGTCAGTAGC CCGGCCATATTCACATAA		501		94 °C/5 min		94 °C/30 s	58 °C/40 s	72 °C/40 s	72 °C/10 min		^83^
*eae*A	ATGCTTAGTGCTGGTTTAGG GCCTTCATCATTTCGCTTTC		248		94 °C/5 min		94 °C/30 s	51 °C/30 s	72 °C/30 s	72 °C/7 min		^84^
Virulence genes used for *Klebsiella pneumonia* isolates^a^												
*rmp*A	ACTGGGCTACCTCTGCTTCA CTTGCATGAGCCATCTTTCA		535		94 °C/5 min		94 °C/30 s	50 °C/40 s	72 °C/40 s	72 °C/10 min		^85^
*Tra*T	GATGGCTGAACCGTGGTTATG CACACGGGTCTGGTATTTATGC		307		94 °C/5 min		94 °C/30 s	55 °C/30 s	72 °C/30 s	72 °C/7 min		^86^
*aerobactin*	GCATAGGCGGATACGAACAT CACAGGGCAATTGCTTACCT		556		94 °C/5 min		94 °C/30 s	50 °C/40 s	72 °C/45 s	72 °C/10 min		^32^
Virulence genes used for *Proteus mirabilis* isolates^a^												
*atp*D	GTATCATGAACGTTCTGGGTAC TGAAGTGATACGCTCTTGCAG		595		94 °C/5 min		94 °C/30 s	58 °C/40 s	72 °C/45 s	72 °C/10 min		^87^
Virulence genes used for *pseudomonas aeruginosa* isolates												
*tox*A	GACAACGCCCTCAGCATCACCAGC CGCTGGCCCATTCGCTCCAGCGCT		396		94 °C/5 min		94 °C/30 s	55 °C/40 s	72 °C/40 s	72 °C/10 min		^88^
*las*B	ACAGGTAGAACGCACGGTTG GATCGACGTGTCCAAACTCC		1,220		94 °C/5 min		94 °C/30 s	54 °C/40 s	72 °C/1 min	72 °C/10 min		^89^
*psl*A	TCCCTACCTCAGCAGCAAGC TGTTGTAGCCGTAGCGTTTCTG		656		94 °C/5 min		94 °C/30 s	60 °C/40 s	72 °C/45 s	72 °C/10 min		^90^
*fli*C	TGAACGTGGCTACCAAGAACG TCTGCAGTTGCTTCACTTCGC		180		94 °C/5 min		94 °C/30 s	56.2 °C/30 s	72 °C/30 s	72 °C/7 min		
Virulence genes used for *acinetobacter baumannii* isolates^a^												
*cnf*1	TATATAGTCGTCAAGATGGA CACTAAGCTTTACAATATTGAC		620		94 °C/5 min		94 °C/30 s	63 °C/40 s	72 °C/30 s	72 °C/10 min		^91^
*iut*A	GGCTGGACATGGGAACTGG CGTCGGGAACGGGTAGAATCG		300		94 °C/5 min		94 °C/30 s	63 °C/30 s	72 °C/45 s	72 °C/7 min		^92^
*cva*C	CACACACAAACGGGAGCTGTT CTTCCCGCAGCATAGTTCCAT		760		94 °C/5 min		94 °C/30 s	63 °C/40 s	72 °C/ 45 s	72 °C/10 min		
Antibiotics resistance genes for all isolates including *Pseudomonas aeruginosa*												
*bla*_*TEM*_	ATCAGCAATAAACCAGC CCCCGAAGAACGTTTTC		516		94 °C/5 min		94 °C/30 s	54 °C/40 s	72 °C/45 s	72 °C/10 min		^93^
*bla*_*SHV*_	AGGATTGACTGCCTTTTTG ATTTGCTGATTTCGCTCG		392		94 °C/5 min		94 °C/30 s	54 °C/40 s	72 °C/45 s	72 °C/10 min		^82^
*bla*_*CTX*_	ATGTGCAGYACCAGTAARGTK ATGGC TGGGTRAARTARGTSACCAGA AYCAGCGG		593		94 °C/5 min		94 °C/30 s	54 °C/40 s	72 °C/45 s	72 °C/10 min		^94^
*qnrs*	ACGACATTCGTCAACTGCAA TAAATTGGCACCCTGTAGGC		417		94 °C/5 min		94 °C/30 s	55 °C/40 s	72 °C/45 s	72 °C/10 min		^95^
*aac*(3)*-Ia*	TTGATCTTTTCGGTCGTGAGT TAAGCCGCGAGAGCGCCAACA		150		94 °C/5 min		94 °C/30 s	55 °C/30 s	72 °C/30 s	72 °C/7 min		^96^
Antibiotics resistance gene for *Pseudomonas aeruginosa* isolate												
*mexR*	GCGCCATGGCCCATATTCAG GGCATTCGCCAGTAAGCGG		637		94 °C/5 min		94 °C/30 s	55 °C/30 s	72 °C/30 s	72 °C/7 min		^97^

The specific sequences that were amplified for each of the used primers (Metabion, Germany).

^a^*fim*H gene was also detected for these isolates.

### Efficacy of *A. nilotica* aqueous extract as an antimicrobial agent

Antibacterial activity of *A. nilotica* aqueous extract against the isolated uropathogens was analyzed by minimal inhibitory concentrations (MIC) by determining the bacterial viability using a colorimetric INT-formazan assay. Thus, we additionally determined the minimal bactericidal concentrations (MBC) which confirmed the killing of the isolated uropathogens over time. The results showed a reproducible and effective antibacterial effect against all isolated uropathogens (Preventing INT color change). Where, the concentration of 11.7 mg ml^−1^ was enough as MIC for all tested organisms except *P. mirabilis* that required a higher concentration of 15 mg ml^−1^. Generally, the efficacy of the extract as bactericidal (MBC) natural product against *E. coli* and *K. pneumoniae* was 13.3 mg ml^−1^. *Pseudomonas aeruginosa* and *A. baumanii* recorded MBC value of 15 mg ml^−1^. Interestingly, *P. mirablis* verified the highest MBC value of 16.7 mg ml^−1^ as shown in Table 3.

### Static biofilm assay

**Figure 4:**
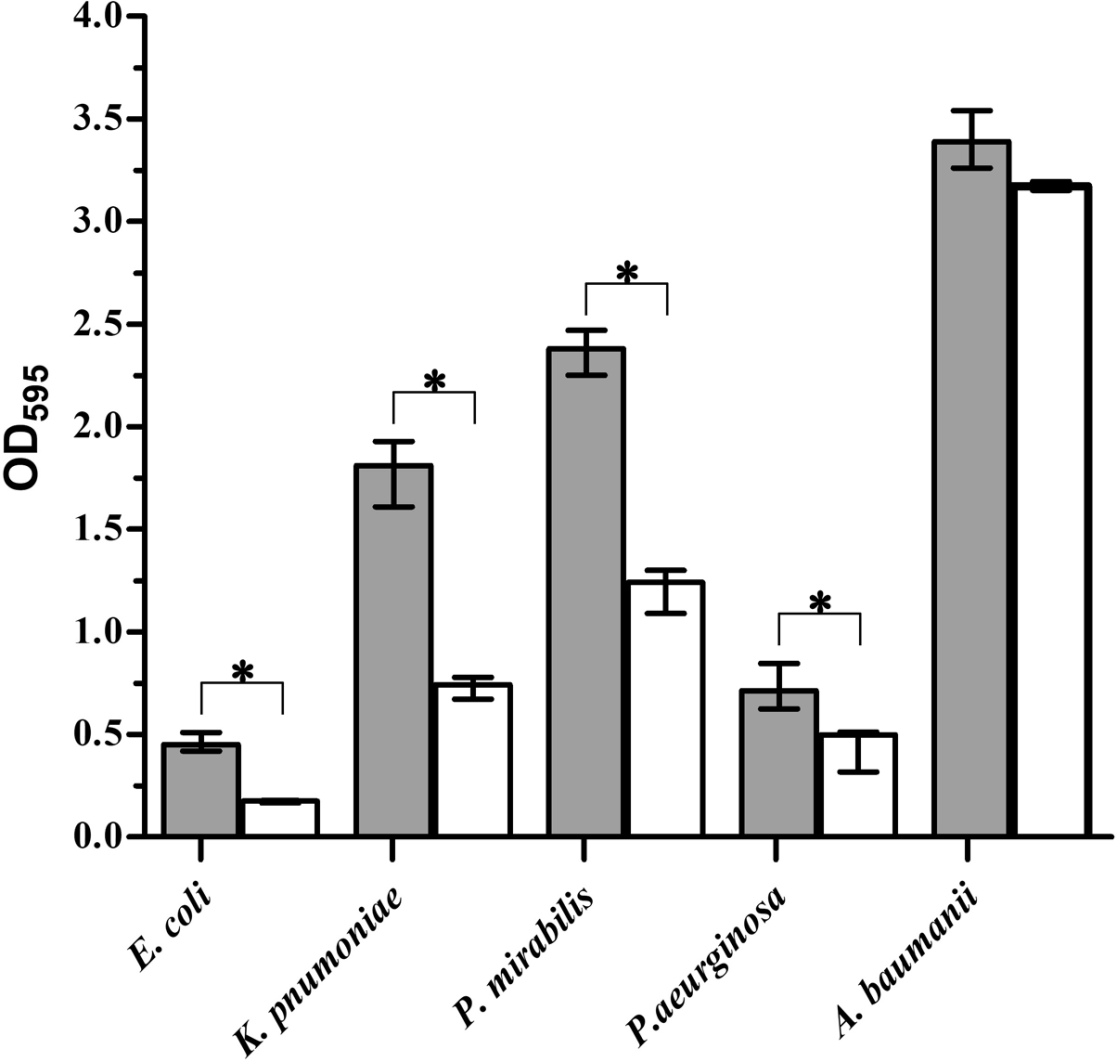
Impact of *Acacia nilotica* aqueous extract on biofilm of some uropathogenic isolates. Quantitative determination of biofilm amount of the isolated uropathogens as control (gray bars) and after treatment with *Acacia nilotica* aqueous extract (open bars). After an additional 24 h biofilm formation was quantified by crystal violet staining and subsequent determination of the OD_595_. Shown are the medians from at least three independent measurements. The error bars indicate the interquartile range. Significant differences between the data sets are marked by asterisks (*P* < 0.05; Kruskal–Wallis test and post hoc Dunn’s multiple comparisons).

Quantitative determination of biofilm amount (OD_595_) of the isolated uropathogens as control and after treatment with *A. nilotica* extract (Fig. 4). According to mean values of OD_595_ nm, the results of control (isolated uropathogens) were interpreted as low, moderate, and high bacterial biofilm former when OD_595_ nm was < 1; 1–2.9 and > 2.9 respectively. Accordingly, *A. baumannii* was a high biofilm former while, *K. pneumoniae* and *P. mirabilis* had a moderate ability. On the other hand, *E. coli* and *P. aeruginosa* were low biofilm former. Interestingly, after treatment with the MBC-values of *A. nilotica* aqueous extract, *E. coli*, *K. pneumoniae*, *P. mirabilis,* and *P. aeruginosa* significantly reduce biofilm by 62.6; 59; 49 and 39.2%, respectively (Fig. 4).

### Survival curve of the isolated uropathogens in the presence of *Acacia nilotica* aqueous extract

**Figure 5:**
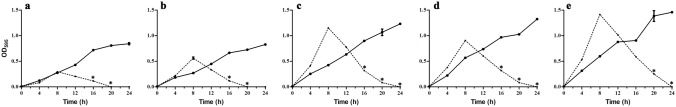
Survival curve of some uropathogenic isolates in the presence of *Acacia nilotica* aqueous extract. Shown are the median OD_595_ values of *Escherichia coli* (**a**); *Klebsiella pneumoniae* (**b**); *Proteus mirabilis* (**c**); *Pseudomonas aeuroginosa* (**d**); and *Acinetobacter baumanii* (**e**) in TSB media (solid line) or supplemented with MBC value of *Acacia nilotica* aqueous extract (dashed line) for each isolate at 37 °C. Shown are the medians from at least three independent measurements. The error bars indicate the interquartile range. Significant differences between the data sets are marked by asterisks (*P* < 0.05; Kruskal–Wallis test and post hoc Dunn’s multiple comparisons).

The killing dynamic of *A. nilotica* aqueous extract against log-phase cultures of the isolated uropathogens was determined to compare with a positive control for each uropathogens (Fig. 5). The MBC-values for the *A. nilotica* aqueous extract were affected by different isolates' growth. The results revealed that after 8 h there is a continuous decrease of all uropathogens cultures OD_595_ with no detectable growth after 20 h exposition for *E. coli* and *K. pneumoniae* (Fig. 5a, b). In the case of *P. mirabilis*, *P. aeruginosa* and *A*. *baumannii* killing curve were also recorded a steady decrease of OD_595_ over time starting after 8 h exposition with no viable microorganism in the initial inoculums could be observed after 24 h. Significant reduction starting at 16 h of treatment for all isolates. Positive control had a continuous increase to 24 h (Fig. 5c–e). Based on these results, Time-kill kinetic profile indicates that *A. nilotica* extracts exhibited bactericidal actions against all uropathogenic isolates (Fig. 5).

## Discussion

UTIs are considered one of the most common groups on infections in humans worldwide that upset kidney, pyelonephritis, bladder, and cystitis^11^. As stated by the CDC, UTIs are the greatest common bacterial infection demanding medical care, resulting in 8.6 million ambulatory care visits in 2007^12^. It is an infection of the urinary tract with a pathogen causing inflammation and occasionally life-threatening^13^. In the current study, although all patients were showing some or all of UTIs symptoms like burning feeling during urination, frequent urge for urination, cloudy appearance of urine, and pain in the back or the lower abdomen^14^. Only about 78.2% of 170 patients had UTIs in this study (Table 1). This is possible because UTI symptoms are not a dependable indicator of disease. So, urine culture is necessary for the diagnosis of UTI for confirming the presence of bacteriuria^14^. The study also verified a lower UTI rate of 7.2% in males comparing with 92.8% in females In agreement with^15^, who stated that UTIs are common in women than men with a ratio of 8:1. A low rate of infection in males may be due to the occurrence of antimicrobial substances in prostatic fluid. Also, maybe a long urethra (20 cm) that provides a distance barrier that eliminates microorganisms from the bladder^16^.

The principal step for effective treatment of UTIs is to classify the type of infection, such as acute uncomplicated cystitis or pyelonephritis, acute complicated cystitis or pyelonephritis, catheter associated-UTI, asymptomatic bacteriuria (ASB), or prostatitis depending on identification of causing organism for describing defective antibiotic^17,18^. About 95% of uncomplicated UTIs are mono-bacterial and *E. coli* is the major causing agent of uncomplicated UTI, which accounts for up to 75–90% of cases^19,20^. This study is in agreement with our study where *E. coli* isolated with a percentage of 73.7% from all isolated uropathogens. *Klebsiella pneumoniae* was the second isolated organism with a percentage of 13.5%, in agreement with^20,21^. Followed by *P. mirabilis* (6.7%), *P. aeruginosa* (4.5%), and *A. baumanii* (1.5%) (Table 1). Also in agreement with^22^, who revealed that uropathogenic *E. coli* (UPEC) is the most common causative agent for both complicated and uncomplicated UTIs, other causative agents are involved like *K. pneumoniae*, *P. mirabilis*, *P. aeruginosa* and other types. Uropathogenic *E. coli* (UPEC) contains several virulence factors that facilitate its colonization and invasion of host cells^23,24^. Surface virulence factors (adhesions) are among the most important virulence factors^25,26^. As the main attachment factor. P fimbriae are particularly associated with pyelonephritis and cystitis which encoded by pap genes^27,28^. Other important virulence factors in UPEC are toxins (secretory virulence factors)^26^. The most important toxin is a-hemolysin (HlyA), which encoded by hly gene that has been detected among pyelonephritis and cystitis^28^. eaeA (intimin or *E. coli* attaching and effacing gene)^29^. Most bacteria regulate a multitude of fimbrial adhesions such as fimbriae 1 type which encoded by fimH gene that was first recognized in *E. coli* ^30^. Our results confirmed the presence of *pap*C, *hly* and *fim*H genes among *E. coli* isolates while, the *eae*A gene was absent among *E. coli* isolates (Table 3; Fig. 1). Several factors contribute to the virulence of *K. pneumoniae* such as the capsular serotype, lipopolysaccharides, iron-scavenging system, and adhesions^31^. These genes include those encoding for regulators of mucoid phenotype A (rmpA) which is detected among local urinary isolates^32,33^. Other *Klebsiella* virulence genes such as type 1 (fimH), type 3 adhesions (mrkD), aerobactin (hydroxamate siderophore which is produced by some enterobacterial strains and TraT gene^34–38^. Our results confirmed the presence of *Tra*T gene and the absence of aerobactin, *fim*H, and *rmp*A genes among *K. pneumoniae* isolates (Table 3; Fig. 1). *Proteus mirabilis* contains several virulence genes that contribute to its pathogenicity such as an *atp*D gene (ATP synthase beta chain)^39^. This gene detected with the percentage of 100% among *P. mirabilis* isolates in our study (Table 3; Fig. 1). Virulence genes in *P. aeruginosa* such as Pilli, exoenzyme S, endotoxin A, and phospholipase C are important for the acute phase of disease while, siderophores and pseudo-capsule of alginate are essential for chronic phase of infections^40^. Elastase (*Las*B gene), phospholipase C, toxin A (*tox*A), and exoenzyme S was assessed in *P. aeruginosa* isolates from UTI^41^ (Table 3; Fig. 1). Our study confirmed the presence of *psl*A gene and absence of *las*B, *tox*A, and *fli*C genes. Some of the most significant virulence genes of *A. baumannii* are colicin V production, curi fibers (*csg*), siderophores like aerobactin (*iut*A), and cytotoxic necrotizing factor (*cnf*)^42,43^. *Acinetobacter baumannii* in our study was free from these genes. However, there is always the possibility of mutation at the level of the corresponding gene, leading to the lack of its detection. Consequently, a positive PCR shows the occurrence of the virulence gene, but a negative PCR does not point to its absence^44–46^ (Table 3).

Biofilm is an accumulation of bacteria reserved within a microbial-derived matrix, which assists their persistence^47^. It contains water passages for transporting oxygen and essential nutrients for growth. Microcolony is the main structural unit of the biofilm it may be composed of 10–25% cells and 75–90% exopolysaccharide (EPS) matrix depending on the species complex^48^. They characterized by a high degree of resistance to antibiotics and host immune defense response substances^4,49^. It also plays an essential role in the pathogenicity of several chronic human infections^50^. In our study, all isolated uropathogens were biofilm former (Fig. 4). Interestingly, the detection of latent virulence genes in the clinical urine isolates also the ability for biofilm formation confirmers the pathogenicity of these isolated uropathogens in the current study. Also has some great epidemiological outcomes to control the dissemination of infectious disease caused by these pathogens. Increasing rates of antibiotic resistance and high repetition rates impend to greatly enhance the problem that these common infections place on society^22^. In a study by^51^, they revealed that antibiotics such as ciprofloxacin and ampicillin are the most commonly recommended therapeutics for UTIs. Interestingly, our study confirmed a great resistance for all isolated uropathogens to ampicillin and ciprofloxacin. In another study by Abuhandan et al.^52^, they reported that all of the isolated uropathogens were resistant to ampicillin-sulbactam, with, high resistance rates recorded for *E. coli* (64.1%) They also stated that the most effective antimicrobial agents were determined to be imipenem, quinolone, and aminoglycosides. It is worth saying that our study showed 100% resistance to ampicillin-sulbactam, 60% resistance to imipenem, 100% resistance to two members of aminoglycosides (Gentamicin and Amikacin) also 100% resistance observed for one member of quinolones (ciprofloxacin) Table 2. This confirms the seriousness of the wrong use of antibiotics over the years. In the current study, the presence of multidrug-resistant genes was determinant such as *bla*_SHV_, *bla*_CTX_, *bla*_TEM_ as it was determined earlier by^53,54^. These genes were detected in our isolates with a percentage of 100, 60, and 60%, respectively (Table 2; Fig. 2). Quinolone resistance is usually resulting from mutations in genes coding for chromosomally-encoded type II topoisomerases, efflux pumps, or porn-related proteins, it also can be plasmid-mediated^55,56^. The plasmid resistance determinants are *qnr*A, *qnr*B, and *qnr*S^56,57^. The *qnr*S gene was detected with percentages of 100% among all isolated uropathogens (Table 2; Fig. 2). Multidrug resistance in *P. aeruginosa* can be caused by regulatory mutations *nal*B (*mex*R), *nfx*B or *nfx*C (*mex*T) leading to overexpression of three separate RND efflux systems which causing multiple antibiotic resistance profiles^58^. In our study *mex*R gene was detected with the percentage of 100% among *P. aeruginosa* isolates (Table 2; Fig. 2). Aminoglycosides resistant genes such as *aac* and *aad*^59^. An example of Gm resistance (*Gmr*) genes was *aac*(3)-Ia^60^. Our results confirmed a 100% resistance to aminoglycosides through the detection of *aac*(3)-Ia gene (Table 2; Fig. 2).

Antibiotic resistance is one of the biggest problems that face the world. Scientists have begun to search for new safe antibiotic alternatives. Medicinal plants are a good substitute for antibiotics^61–66^. The pods of *A. nilotica* extract was good antibacterial agent against different bacterial pathogens^64^. Our study confirmed the greatest efficacy of *Acacia nilotica* extract against all isolated uropathogens with MBC of 15–16.7 mg ml^−1^ with the greatest MBC value obtained by *P. mirabilis* (Table 3). The analysis of time killing data confirmed that *A*. *nilotica* aqueous extract kills *E. coli* and *K*. *pneumoniae* (within 20 h) faster than other uropathogens (Fig. 5). *Acacia nilotica* extract also reduces the biofilm of the tested pathogens (Fig. 4). This is could be due to the presence of some active phytochemicals such as 3-Cyclohexane-1-Carboxaldehyde, 2,6,6-trimethyl; á-Selinene; Oleic Acid; Globulol and Isochiapin that were detected in the GC–MS analysis (Table 4; Fig. 3). The antibacterial activity of crude extracts and different fractions could be largely due to the effect of the phytochemicals detected^67^. In study by^68^, the phytochemical analysis of *A. nilotica* pod extracts by LCMS, HPLC/DAD, and FTIR was confirmed as antibacterial agents against antibiotic-resistant strains of *E. coli* and *Salmonella* sp. Cyclohexane, for example, is considered the most potent antibacterial agent that had a reduced ability to inhibit solute transport in comparison with other active analogs^69^. The oleic acid produced by marine spp. also could be valuable as a biocontrol against gram-negative bacteria including *Vibrio parahaemolyticus* and might denote an influence in the clinical use^70^. Other important phytochemical components detected with a high percentage in *A. nilotica* aqueous extract such as á-Selinene; Globulol and Isochiapin were also recorded for their antibacterial activities^71–73^.

In conclusion, a new preventive measure against multidrug-resistant isolated uropathogens which confirmed by multiplex PCR, consists of the use of *A. nilotica* aqueous extract. *Acacia nilotica* considered a natural antimicrobial agent to prevent bacterial growth, biofilm formation, and decreases the dissemination of these multidrug-resistant strains. However, optimizing the production of the active organic products of *A. nilotica* extract is a challenge that must be considered to use this compound to contrast the pathogenic action of UTIs. Also, it is recommended to make purification of *A. nilotica* extract to test one or more of the larger concentration of some compounds like 3-Cyclohexane-1-Carboxaldehyde, 2,6,6-trimethyl; á-Selinene (CAS); Oleic Acid; Globulol in the composition of the extract against uropathogens and performing in vivo experiments.

## Materials and methods

### Ethical approval and informed consent

The study protocol was approved by the local Medical Ethics Committees of the Medical University of Assiut, Egypt, which has been approved by the Egyptian Ministry of Higher Education and Scientific Research on 11/2009. General hospital and private medical analysis laboratories in Luxor province, Egypt ethically approved urine sampling and informed consent was obtained from all participants during the study work. The methods were carried out in accordance with the relevant guidelines and regulations, and the subjects gave written informed consent.

### Sampling; isolation and identification of uropathogens

A total of one hundred and seventy urine samples were collected between January to June (2019) from the general hospital and private medical analysis laboratories in Luxor province, Egypt. Patients were between 8 and 86 years old, they were 110 females and 60 males. Urine samples were collected by clean catch mid-stream urine collection method into the sterile container from patients who had not received antimicrobials within the previous one week. Guidelines for proper specimen collection were given to all patients on a printed card^74^. All samples were subjected to COMISCREEN 10 SL urine test strips for a rapid- semi-quantitative determination of leucocyte (pyuria) using a leucocyte esterase test (LET) and nitrite to detect bacteriuria. Samples were examined using a light microscope/high-power (HPF) (LEICA DMLF2, China) for the presence of 10 or more white blood cells^75^. For isolation of uropathogens, samples were streaked onto MacConkey (OXOID), Eosin methylene blue (BIOWORLD, USA), and Tryptic soy agar (OXOID) plates then incubated at 37°C for 24h. Isolates were picked up and identified by standard biochemical methods^76–78^. CFU/ml (colony forming units) were also determined.

### Antimicrobial sensitivity testing

The antibiograms for all the recovered isolates were determined as described earlier according to the Kirby Bauer disk diffusion method^79^. The susceptibility of all isolates was tested for 12 antibiotics from different groups (BIOANALYZE). The used antibiotics were Imipenem (10 μg), Meropenem (10 μg), Ciprofloxacin (5 μg), Ceftazidime (30 μg), Amikacin (30 μg), Nalidixic acid (30 μg), Gentamicin (10 μg), Ampicillin (10 μg), Ampicillin-sulbactam (10/10 μg), Piperacillin (100 μg), Piperacillin-tazobactam (100/10 μg) and Cefepime (30 μg). Interpretation of the results was performed according to clinical and laboratory standard institute guidelines^80^ to determine if the isolate is resistant, intermediate, or susceptible to the tested antibiotics.

### Detection of virulence and antibiotic-resistant genes of isolated uropathogens

Molecular characterization of the recovered uropathogens was carried out by multiplex PCR. The detected enterotoxins genes for *E. coli* were (*fim*H, *pap*C, *hly,* and *eae*A). For *K. pneumonia* (*fim*H, *rmp*A, *Tra*T and aerobactin), for *P. mirbilis* (*fim*H and *atp*D), for *P. aeruginosa* (*tox*A, *las*B, *fli*C, and *ps*lA), for *A. baumannii* (*fim*H, *Cnf*1, *iut*A and *cvaC*). While the detected antibiotic-resistant genes for all isolates were *bla*_TEM_, *bla*_SHV_, *bla*_CTX_, *aac*(3)*-Ia,* and *qnr*s. Besides, the *mexR* gene was performed for *P. aeruginosa* only. The encoding enterotoxins and antibiotic-resistant genes (twenty-one) were performed using (forty-two) primers sets including forward and reverse. All primer sequences with corresponding references are listed in Table 5^32,81–97^.

### DNA amplification for the selected virulence and antibiotic resistance genes of isolates

The extraction of DNA was carried out according to QIAamp DNA mini kit instructions (QIAGEN, Germany, GmbH) as described earlier by Bisi-Johnson^21^ with modification from the manufacturer's recommendations. Briefly, 200 μl of the sample suspension was inoculated with 10 μl of proteinase K and 200 μl of lysis buffer at 56 °C for 10 min. After incubation, 200 μl of 100% ethanol was added to the lysate. The sample was then washed and centrifuged following the manufacturer's recommendations. Nucleic acid was eluted with 100 μl of elution buffer provided in the kit. PCR amplification was performed using oligonucleotide primer (METABION, Germany) that were utilized in a 25 μl reaction containing 12.5 μl of EMERALDAMP Max PCR Master Mix (TAKERA, Japan), 1 μl of each primer of 20 pmol concentration, 5.5 μl of dist. water and 6 μl of DNA template. The reaction was performed in an applied biosystem 2,720 thermal cycler. All primers amplicon sizes and cycling conditions are summarized in Table 5^32,81–97^. The products of PCR were separated by electrophoresis on 1.5% agarose gel (APPLICHEM, Germany, GmbH) in 1xTBE buffer at room temperature using gradients of 5 V/cm. For gel analysis, 15 μl of the products were loaded in each gel slot. Gelpilot 100 bp and 100 bp plus ladders (QIAGEN, Germany, GmbH) and GeneRuler 100 bp ladder (FERMENTAS, THERMO) was used as a marker for electrophoresis to determine the fragment sizes. The gel was photographed by a gel documentation system (ALPHA INNOTECH, BIOMETRA) and the data was analyzed through computer software (AUTOMATIC IMAGE CAPTURE, USA).

### Gas chromatography-mass spectrometer (GC–MS) analysis

GC–MS technique was used in this study as described earlier by Sadiq et al.^68^, to identify the Phyto-components present in the plant extract. For preparing *A. nilotica* aqueous extract, 20 gm of dry pods were ground into fine powder by using an electric grinder (SOGO, China). The powder was soaked in 100 ml of hot distilled water and then cooled down with continuous stirring at room temperature by using bigger bill shaker, USA, for extraction of active ingredients^98^. The mixture was filtered then sterilized using a syringe filter equipped with a 45μ membrane filter; then kept at 4 °C. *Acacia nilotica* material was subjected to gas chromatography-mass spectrometer technique (GC–MS) (THERMO SCIENTIFIC TECHNOLOGIES, TRACE 1,310) with capillary column TG-5 (30 m × 250 μm × 0.25 μm) system were used. The mass detector used in split mode and helium gas with a flow rate of 1.5 ml/min was used as a carrier. The injector was operated at 230 °C and the oven temperature for the initial setup was 60 °C for 2 min. ramp 10/min. to 300 °C for 8 min. Mass spectra were taken at 70 eV, total GC running time was 35 min.

### Determination of the minimum inhibitory concentration (MIC) and minimum bactericidal concentration (MBC) by INT reduction assay

The determination of MIC and MBC were assayed as described by^99^. Where the freshly prepared culture of isolated uropathogens was adjusted to OD_595_ of 0.01. 100 μl of each bacterial fresh culture was put into sterilized 96-well plates. Then 20 μl of the original extract was added (serial dilutions of 10^−1^–10^−10^ were used, 8 replicates were made for each dilution into complete raw of the 96-well plate). The plates incubated at 37 °C for 24 h. MIC was determined by the addition of 40 μl of *p*-iodonitrotetrazolium violet chloride (INT) (0.2 mg/ml, SIGMA-ALDRICH) to the plates and re-incubated at 37 °C for 30 min., the lowest concentration which banned color change is the MIC^100,101^. MBC was determined according to to^99,102^.

### Static biofilm assay

The recovered uropathogenic isolates were assessed for their biofilm activity in a microtiter plate according to to^103^ after modifications by^104^ as follows: Isolates were grown on TSA for 24 h at 37 °C, suspended in TSB, adjusted to an OD_595_ of 0.02. Then, 130 μl from each isolate culture were plated into a 96-well microtiter plate (U BOTTOM, STERILIN) for 24 h at 37 °C. Then for studying the antibiofilm activity of the extract, 30 µl of the *A. nilotica* aqueous extract—(MBC) value for each isolate—was added After 24 h. The addition of 30 µl of sterilized H_2_O to the original biofilm of the isolated uropathogens served as control. Wells were consequently rinsed with H_2_O and the biofilm was stained with 0.1% crystal violet, solubilized in 96% ethanol, and the OD_595_ was measured using INFINITE F50 ROBOTIC (Ostrich) Microplate Reader to quantify the amount of biofilm. Each treatment was added to three wells i.e. three replicates.

### Survival curve of the isolated uropathogens in the presence of *A. nilotica* aqueous extract

An increase, both in total cell mass and cell number can readily be estimated by measuring the turbidity of a cell suspension using an instrument such as a spectrophotometer^105^. So, the microbial population at the initial and completion of the experiment isolates were grown overnight on TSA plates, suspended in TSB to an OD_595_ of 0.01 then incubated with the MBC value of *A. nilotica* aqueous extract for each isolate at 37 °C. Adjusted culture from each isolated uropathogens at OD_595_ of 0.01 served as the positive control. Approximately, 1 ml aliquot was tested from the culture medium over time (0, 4, 8, 12, 16, 20, and 24 h) for monitoring the optical density of all bacterial treatments at OD_595_ nm using the ‘ ‘SPECTRONIC GENESYS 2PC” Spectronic Instruments, USA. Readings were taken three times. Results were confirmed by taking 50 µl of each treatment at OD_595_ of 0.0 (complete killing) onto fresh TSA and incubation at 37 °C for 24 h (Three plates were used for each isolate).

### Statistical data analysis

Data were analyzed using the Mann–Whitney U test or a Kruskal–Wallis test followed by post hoc Dunn’s multiple comparisons. Differences were considered significant at *P* values of ≤ 0.05. For all statistical analyses, GraphPad Prism version 5 was used.
